# Simultaneous profiling of transcriptome and DNA methylome from a single cell

**DOI:** 10.1186/s13059-016-0950-z

**Published:** 2016-05-05

**Authors:** Youjin Hu, Kevin Huang, Qin An, Guizhen Du, Ganlu Hu, Jinfeng Xue, Xianmin Zhu, Cun-Yu Wang, Zhigang Xue, Guoping Fan

**Affiliations:** Department of Human Genetics, David Geffen School of Medicine, UCLA, 695 Charles Young Drive South, Los Angeles, CA 90095 USA; Translational Center for Stem Cell Research, Tongji Hospital, Department of Regenerative Medicine, Tongji University School of Medicine, Shanghai, 200065 China; Division of Oral Biology and Medicine, Laboratory of Molecular Signaling, University of California Los Angeles, Los Angeles, CA 90095 USA; Suzhou Institute, Tongji University, Suzhou, Jiangsu Province 215101 China

**Keywords:** Single-cell methylome, Single-cell transcriptome, Sensory neurons, Dorsal root ganglion, Gene regulation

## Abstract

**Background:**

Single-cell transcriptome and single-cell methylome technologies have become powerful tools to study RNA and DNA methylation profiles of single cells at a genome-wide scale. A major challenge has been to understand the direct correlation of DNA methylation and gene expression within single-cells. Due to large cell-to-cell variability and the lack of direct measurements of transcriptome and methylome of the same cell, the association is still unclear.

**Results:**

Here, we describe a novel method (scMT-seq) that simultaneously profiles both DNA methylome and transcriptome from the same cell. In sensory neurons, we consistently identify transcriptome and methylome heterogeneity among single cells but the majority of the expression variance is not explained by proximal promoter methylation, with the exception of genes that do not contain CpG islands. By contrast, gene body methylation is positively associated with gene expression for only those genes that contain a CpG island promoter. Furthermore, using single nucleotide polymorphism patterns from our hybrid mouse model, we also find positive correlation of allelic gene body methylation with allelic expression.

**Conclusions:**

Our method can be used to detect transcriptome, methylome, and single nucleotide polymorphism information within single cells to dissect the mechanisms of epigenetic gene regulation.

**Electronic supplementary material:**

The online version of this article (doi:10.1186/s13059-016-0950-z) contains supplementary material, which is available to authorized users.

## Background

DNA methylation involves the covalent attachment of a methyl group to the fifth carbon of cytosine. It is thought that such a modification plays a critical role in regulating gene expression for tissue- and cell-specific transcriptional programs [1–3]. The current model suggests that promoter methylation stably silences gene expression, particularly in the regulation of developmental and tissue-specific gene expression [4]. However, most of the previous studies analyze the correlation of DNA methylation with gene transcription in bulk cell populations. It is still unclear whether variations of gene expression at the single-cell level can be explained by differential methylation at individual gene promoters. In fact, it would be necessary to integrate methylome and transcriptome analysis in a single cell to provide a direct connection between DNA methylation and gene transcription at a given gene locus [5–8].

In recent years, we have seen the rapid development of single-cell genomics methods such as single-cell RNA sequencing (RNA-seq) [9–11], single-cell bisulfite sequencing (BS-seq) [12], and single-cell reduced representation bisulfite sequencing (RRBS) [13] to profile transcriptome and DNA methylome at the genome scale. These studies have revealed important biology with regards to cellular heterogeneity and developmental mechanisms [11, 14–17]. To further understand the correlation of DNA methylation and transcriptome within the same cell, we developed a simultaneous single-cell methylome and transcriptome sequencing (scMT-seq) method, in which cytosolic RNA is isolated for RNA-seq whereas genomic DNA from the same nucleus is subject to DNA methylome profiling. Our study uncovered complex relationships between gene expression and DNA methylation in proximal promoter and gene body regions within a single cell.

## Results

### Cytosol transcriptome resembles the whole-cell transcriptome

**Fig. 1:**
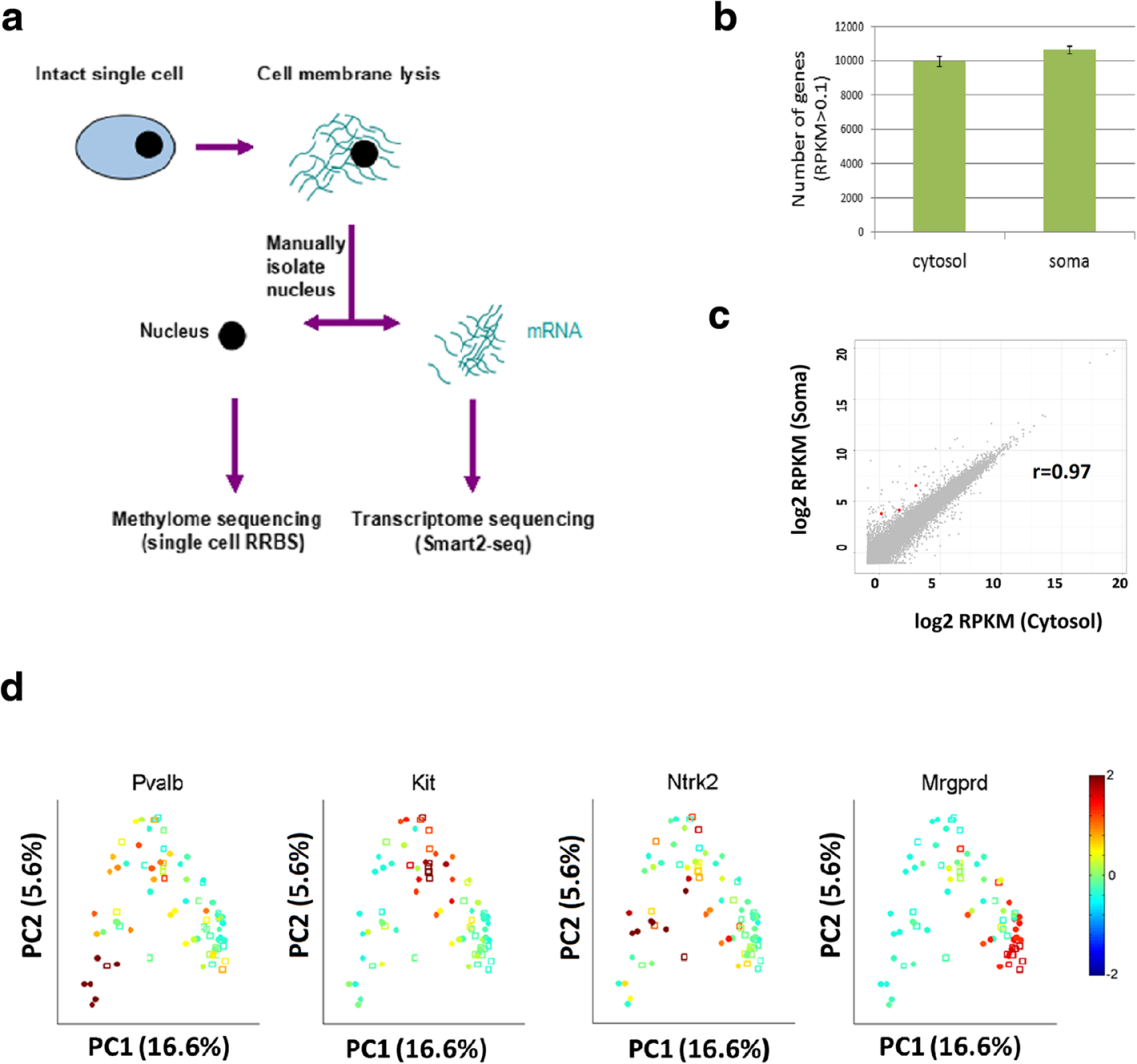
Single-cell cytosol transcriptome resembles single-soma transcriptome. **a** *Schematic* of the single-cell transcriptome and methylome sequencing (scMT-seq) method. **b** Comparison of single-cell cytosol RNA-seq and soma RNA-seq in terms of the coverage of gene number. Only genes with reads per kilobase per million (RPKM) >0.1 were counted. **c** *Scatter plot* of transcript expression levels in cytosol (*x-axis*) or soma (*y-axis*) samples. *Red dots* indicate the significantly differentially expressed genes (*p* <0.01) and *gray dots* indicate genes that are not differentially expressed. **d** Principal component analysis for DRG single soma and cytosol RNA-seq libraries. The relative expression levels of known marker genes for specific subgroups are shown in color. *Red* represents high expression while *blue* represents low expression. *Solid circles* represent cytosol; *empty squares* represent soma

To study the transcriptome of cytosolic RNA from a single cell, we performed single-cell RNA-seq from individual sensory neurons isolated from adult mouse dorsal root ganglion (DRG). These cells tend to be large (20–50 microns in diameter) and enable facile micro-manipulation. Briefly, adult mouse DRG was freshly dissected and dissociated into single cells, then individually transferred to a droplet of cell membrane lysis buffer. Since the lysis buffer does not lyse the nuclear membrane, the cytosolic fraction can be manually separated from nucleus by micropipette manipulation. The cytosolic fraction was then subjected to transcriptome profiling via the Smart2-seq protocol [18], while the isolated nucleus was subjected to methylome analysis by using a modified single-cell RRBS protocol [19] (Fig. 1a).

To control for technical variations in the micro-pipetting technique, we performed a “merge-and-split” experiment for nine pairs of single-cell cytosolic RNA. Principal component analysis (PCA) indicated that each of the “merged-and-split” pair share greater similarity within the pair than with other pairs (Additional file 1: Figure S1A). Furthermore, technical variation was assessed by analyzing the consistency of amplified ERCC RNAs that were spiked into scRNA-seq libraries. The Pearson correlation of ERCC RNAs among different cells were highly similar (r >0.88) (Additional file 1: Figure S1B).

With the technical assurance aside, we generated RNA-seq libraries from 44 cytosol and 35 single soma samples that were sequenced with an average of 2 million reads per sample. We found that cytosol RNA-seq and soma RNA-seq detected 9947 ± 283 and 10,640 ± 237 (mean ± SEM) genes respectively (Fig. 1b). Moreover, by computing the coefficient of variance as a function of read depth for each gene, we found that cytosol and soma exhibit nearly identical levels of technical variation across all levels of gene expression (Additional file 1: Figure S2).

Consistently, Pearson correlation analysis showed that the transcriptome of cytosolic RNA is highly correlated with RNA from the soma (r = 0.97, Fig. 1c). Differential expression analysis showed only 3 out of 10,640 genes (0.03 %) were significantly different between cytosol and soma (false discovery rate [FDR] <0.01), including *Comp*, *Serpina3i*, and *A330023F24Rik*. PCA clustering revealed that all samples clustered into four major subgroups, consistent with previous subclassification of sensory neurons [11]. For example, DRG cells were positive for different marker genes of various neuronal subtypes such as: (1) peptidergic (*Kit* positive); (2) non-peptidergic (*Mrgprd* positive); (3) low threshold mechanoreceptors (*Ntrk2* positive); and (4) proprioceptive (*Pvalb* positive) neurons (Fig. 1d). Cytosol and soma samples were found evenly distributed across the four major clusters without any apparent biases, further indicating that the transcriptome of cytosol and soma are highly similar. Together, these results demonstrate that the cytosolic transcriptome can robustly represent the soma transcriptome.

### Simultaneous DNA methylome analysis in conjunction with single-cell cytosol RNA-seq

**Fig. 2:**
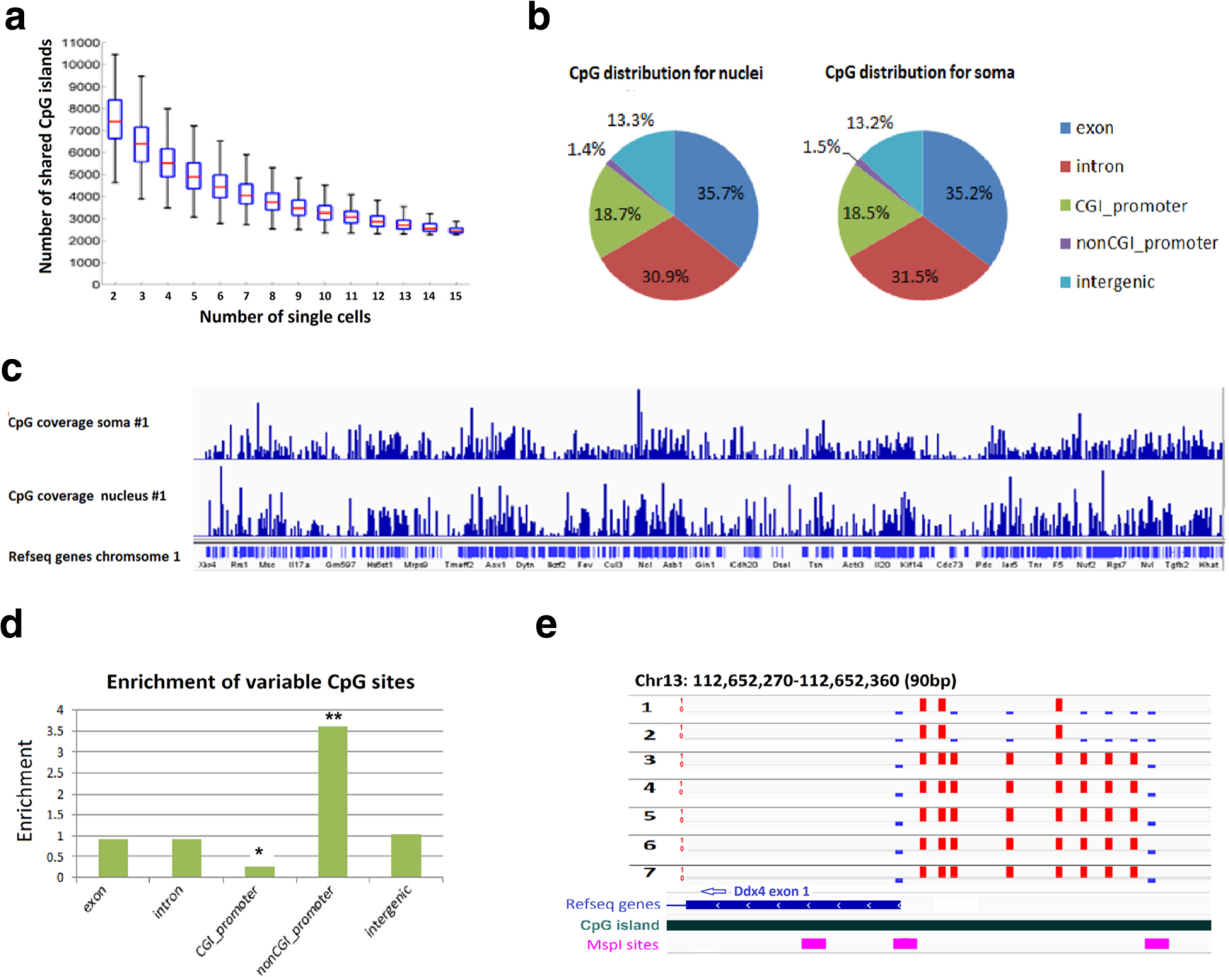
DNA methylome analysis of single DRG neuronal nucleus. **a** *Boxplots* showing the distribution of overlapping CGIs between randomly sampled number of cells as indicated on the *x-axis*. **b** *Pie chart* with the genomic distribution of all CpG sites detected in nucleus and soma RRBS libraries. **c** Genome *browser tracks* showing the coverage of CpG sites for chromosome 1 that are covered by soma methylome (*top*) or nucleus methylome (*bottom*). **d** *Bar graph* showing the genomic features that are enriched for differentially methylated CpG sites across scRRBS libraries. * and ** indicate differential distribution of differentially methylated CpG sites at CpG island promoter and non-CpG island promoter region, respectively (*p* <10^−8^, binomial test). **e** The heterogeneous methylation status of a representative locus at promoter region of Ddx4. *Red bars* indicate the methylated CpG sites, *blue bars* indicate the unmethylated CpG sites

In parallel to cytosol RNA-seq, we extracted DNA from the nucleus of the same cell and performed methylome profiling using a modified single-cell RRBS (scRRBS) method [13]. On average, we sequenced each sample to a depth of 6.7 million reads, which is sufficient to calculate the vast majority of CpGs as indicated by saturation analysis (Additional file 1: Figure S3). Bisulfite conversion efficiency was consistently greater than 99.4 % as estimated by analyzing conversion of unmethylated spike-in lambda DNAs (Table 1). The average number of CpG sites assayed per single nucleus was 482,081, in the range of 240,247–850,977 (Table 1). In addition, we examined the CpG islands (CGI) coverage as RRBS is biased for covering regions rich in CpG sites. *In silico* digestion revealed that 14,642 out of all possible 16,023 CGI (91 %) in the mouse genome can be covered by at least one RRBS fragment. In our experiments, we found that each cell can cover an average of 65 % CGIs, in the range of 50–80 %. Between any two single cells, the median number of shared CGI covered is 7200. Moreover, about 3200 CGIs are commonly covered between 15 libraries (Fig. 2a). Together, these data indicate a high concordance of coverage for CGI.

**Table 1:** Simultaneous sequencing of single-cell methylome and transcriptome

Samples	Mouse strain	CpG (1×)	CpG (5×)	Conversion rate	Promoter no. methylome^a^	Gene no. intersected with transcriptome^a^
sc-1#	129/B6	495,313	342,121	99.59 %	4930	3627
sc-2#	129/B6	362,211	238,120	99.57 %	3905	2901
sc-3#	129/B6	909,730	498,910	99.64 %	6305	4658
sc-4#	129/B6	850,977	547,785	99.48 %	6059	4457
sc-5#	129/B6	565,739	350,744	99.77 %	5234	3911
sc-6#	129/B6	442,073	293,706	99.63 %	4370	3235
sc-7#	129/B6	413,412	231,743	99.94 %	3714	2759
sc-8#	129/B6	240,247	173,079	99.85 %	3106	2315
sc-9#	B6/D2	379,925	233,773	99.55 %	4272	3164
sc-10#	B6/D2	549,887	257,318	99.62 %	4263	3141
sc-11#	B6/D2	437,273	221,168	99.38 %	3675	2708
sc-12#	B6/D2	434,886	183,527	99.57 %	3388	2501
sc-13#	B6/D2	368,404	132,999	99.36 %	2792	2113
sc-14#	B6/D2	456,637	242,423	99.67 %	4032	3015
sc-15#	B6/D2	474,163	220,723	99.49 %	3902	2885

^a^The gene numbers are calculated by CpG coverage of 5× at promoter region

Coverage comparisons between the single DRG nucleus methylome and the single DRG soma methylome did not reveal any substantial differences (Fig. 2b, c, and Additional file 1: Table S1). Both nucleus and soma methylomes could cover on average approximately 277,000 CpG sites (> = 5 reads), which is similar to data generated from a previous report describing scRRBS [13] (Additional file 1: Table S1). As expected, nuclear and soma methylomes are by and large equivalent.

To study methylation heterogeneity among single cells, we first examined CpG sites that were differentially methylated among individual cells*.* As RRBS predominantly covers regions of high CG density which are frequently hypomethylated, it is expected that no difference would be found in the majority of CGs in CGIs. However, by examining the variance of individual CpG sites that were shared in at least 50 % of the samples (n >8), we identified ~6800 CpG sites that were significantly variable (FDR <1 %, F-test, Additional file 1: Figure S4). Genomic annotation of these differentially methylated CG sites revealed a 3.6-fold enrichment at non-CGI promoters and a 3.8-fold depletion at CGI promoters compared to the background of total CpG sites tested (*p* <10^−8^, binomial test, Fig. 2d, Additional file 1: Figure S5). While this result suggests that CpG methylation in non-CGI regions significantly contributes to the methylome variation between cells, we also found differential DNA methylation in individual CGIs in adult DRG neurons. Fig. 2e shows a representative locus with differential methylated CpG sites at CGI promoter region of *Ddx4.* Among ten CpG sites with this region, three CpGs were found to be fully methylated while two were fully unmethylated among all seven neurons. The remaining five CpG sites were methylated in five neurons but unmethylated in two other neurons. Taken all together, our single-cell methylome analysis uncovered regions of methylation heterogeneity among individual DRG neurons.

### Correlation of proximal promoter DNA methylation with gene expression in a single neuron

**Fig. 3:**
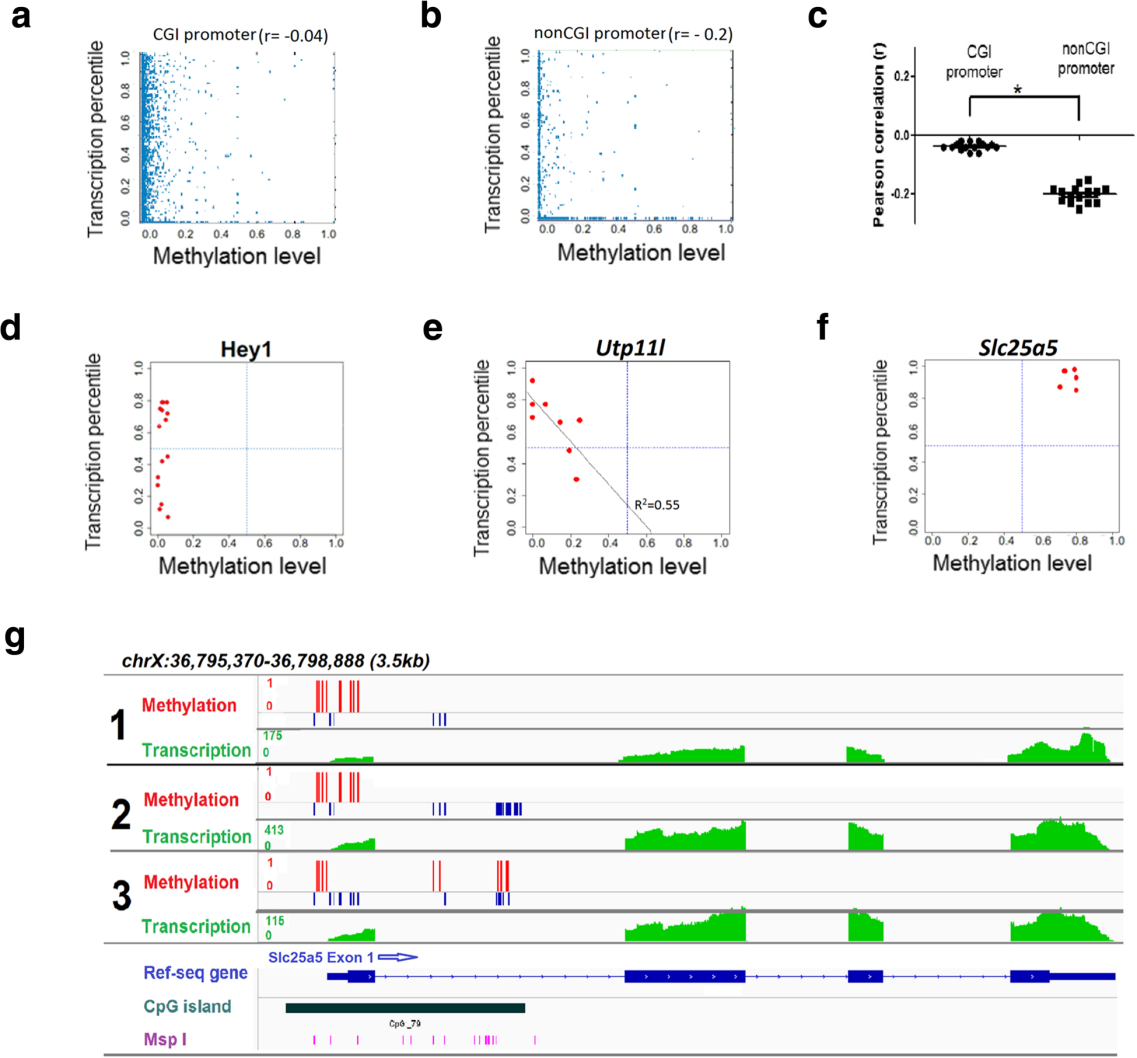
Simultaneous profiling of promoter methylation and gene expression from a single neuron. **a** Representative *scatter plot* for CGI promoter methylation level and transcription level of genes at whole genome wide within a representative single cell. Promoter methylation level was calculated by the ratio of methylated CpG sites over all CpG sites within the promoter region. Expression level was transformed to expression percentile. **b** Representative *scatter plot* for non-CGI promoter methylation level and transcription level of individual genes within a representative single cell. **c** *Dot plot* of Pearson correlation coefficients between transcription level (as expression percentile) and promoter methylation. **d** Representative example of genes with hypomethylation promoter and dynamic expression. Each *point* represents a single cell. **e** Representative example of genes that differential promoter methylation is negatively correlated with gene expression. Each *point* represents a single cell. **f** Representative example of genes with hypermethylation promoter and high expression. Each *point* represents a single cell. **g** Genome *browser tracks* for *Slc25a5* showing promoter hypermethylation and high gene expression in three representative male single cells. *Red bars* indicate the methylation CpG sites and *blue bars* indicate the unmethylated CpG sites. RNA transcription level is shown in *green*. The CpG island reference and MspI cut sites are in *dark green* and *purple*, respectively. *p <0.0001

Integrated analysis of the nuclear DNA methylation and the cytosolic RNA datasets provides us the unique opportunity to investigate the genome-wide correlation of methylation and transcription in the same cell. Among the 4263 ± 258 (mean ± SEM; n = 15) promoters analyzed for both DNA methylation and RNA transcription, we found messenger RNA (mRNA) transcripts in 3159 ± 189 (74.2 %) genes (reads per kilobase per million [RPKM] >0.1) (Table 1). The remaining fraction of genes (~26 %) are either silenced or expressed at very low levels (RPKM <0.1). Overall, consistent with previous findings, our data indicated that promoter methylation is negatively correlated with gene expression (Additional file 1: Figure S6). However, by subclassifying promoters into CGI versus non-CGI promoters, we found that most of the negative correlation is driven by non-CGI promoters. CGI promoters are predominantly hypomethylated and have no predictive power on gene activity (Pearson = −0.05) (Fig. 3a, Additional file 1: Figure S7). By contrast, methylation of non-CGI promoters showed a stronger anti-correlation with transcriptional activity (Pearson = −0.22) (Fig. 3b, c, Additional file 1: Figure S8).

We next examined the correlation of promoter methylation with gene transcription for individual genes across cells. For those hypomethylated gene promoters, we found that 49 % of genes are expressed at similar levels across all cells, consistently either low or highly expressed as represented by *Zfp609* and *Rps18* (Additional file 1: Figure S9). On the other hand, the other 51 % of hypomethylated genes exhibit dynamic expression across cells (i.e. genes that exhibit differential gene expression irrespective of gene promoter methylation). For example, *Hey1* gene promoter is constitutively hypomethylated but is highly expressed in 7/14 (50 %) cells and low expressed in the other seven cells (50 %) (Fig. 3d). Together, these data suggest that other factors are involved in regulating genes with hypomethylated promoters.

We next took a reverse approach and examined genes with promoters that were variably methylated between single cells. In total, we identified 23 gene promoters that were variably methylated, six (26.1 %) of which were significantly correlated with gene transcription (*p* <0.05, Fisher’s Transformation). These genes include *Utp11l*, *Ubl4*, and *Atg13* (Fig. 3e). Interestingly, we identified a rare subset of CpG rich gene promoters that are hypermethylated but still highly expressed. For instance, the X chromosomal linked gene *Slc25a5*, a member of the mitochondrial carrier subfamily of solute carrier protein genes, shows high and robust expression despite a fully methylated promoter. There is no clear evidence for any alternative promoters or neighboring genes that could explain the high expression (Fig. 3f). However, we still observed the CpG hypermethylation around the TSS and the high expression of this gene (Fig. 3g). Collectively, these data paint a complex picture for the role of promoter methylation in gene regulation.

### Correlation of gene body methylation with gene expression

**Fig. 4:**
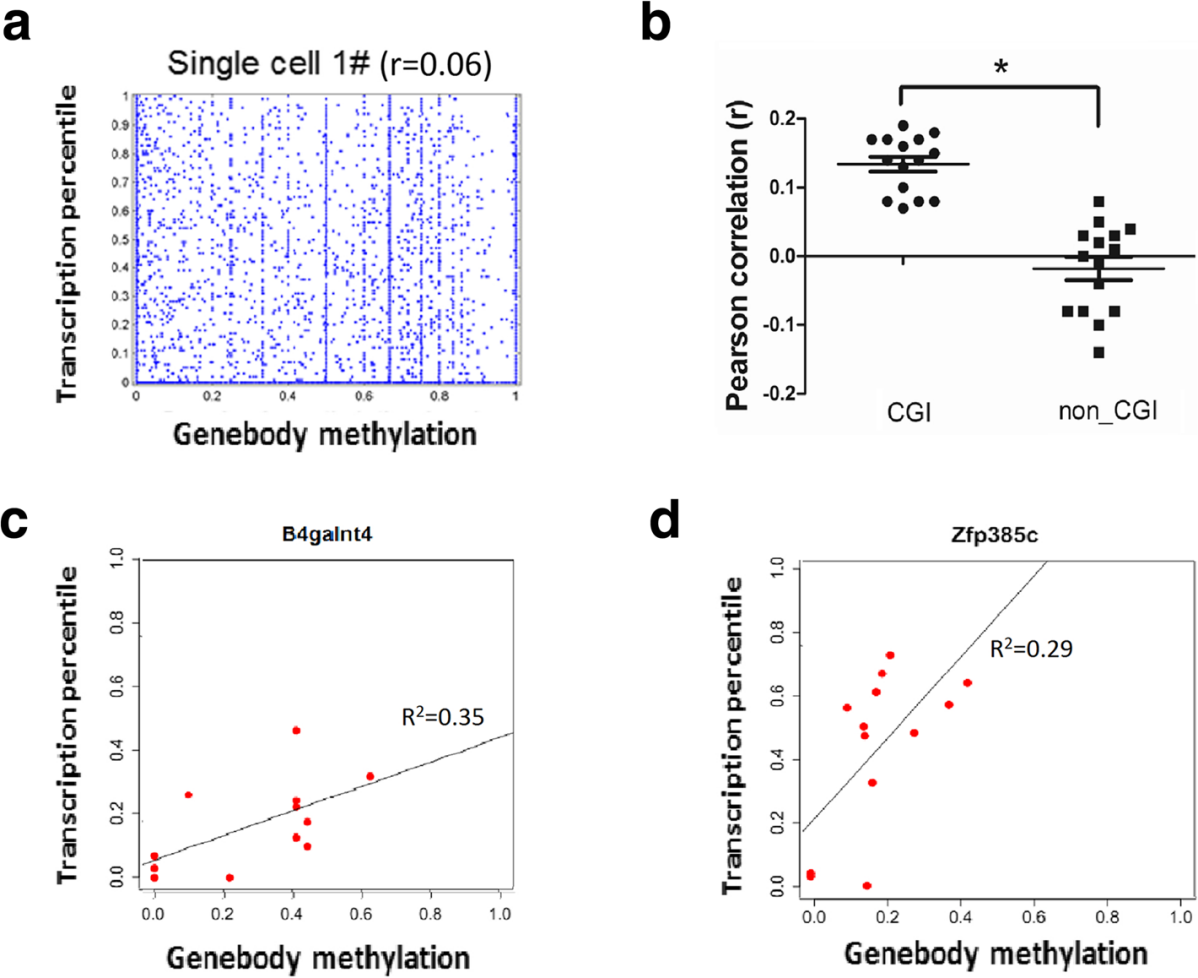
Correlation of gene body methylation with gene expression in a single neuron. **a** *Scatter plot* of gene body methylation and transcription level for genes within single neuron cells. **b** *Dot plot* of Pearson correlation coefficients between transcription level (as expression percentile) and gene body methylation. The genes with CpG sites detected in the region more than 0.5 Kb were clustered into two groups, CGI promoter genes and non-CGI promoter genes. **c**, **d** Representative *scatter plot* examples of the CGI promoter genes which are expressed and positively correlated with gene body methylation. *p <0.0001 (Student’s *t*-test)

Unlike promoter methylation, gene bodies show a wide spectrum of methylation in individual cells (Fig. 4a, Additional file 1: Figure S10). However, the role of gene body methylation is not well studied. As a whole, gene body methylation tends to positively correlate with gene expression (Pearson = 0.06, Fig. 4a). By further subclassifying gene bodies by their promoter (either CGI or non-CGI promoters), we found that gene body methylation is positively correlated with CGI promoter genes (*r* = 0.13), but not with non-CGI promoter genes (Fig. 4b). Furthermore, we examined the correlation of methylation with transcription for 606 genes with differential gene body methylation level between single cells. Transcription of 29 genes (4.8 %) were found to be positively correlated with changes in gene body methylation, 65.5 % of which were CGI promoter genes such as *B4galnt4*, *C1qtnf4*, *Ccdc9*, *Clasrp*, *Jag2*, *Mxra7*, *Tcf3*, and *Trib2* (Fig. 4c, d). Together, these results indicate that gene body methylation would be a better indicator of gene transcription levels compared to promoter methylation for CGI promoter genes. By contrast, proximal promoter would be a better indicator of gene transcription for non-CGI promoter genes.

### Profile of allelic-specific transcription and methylation

**Fig. 5:**
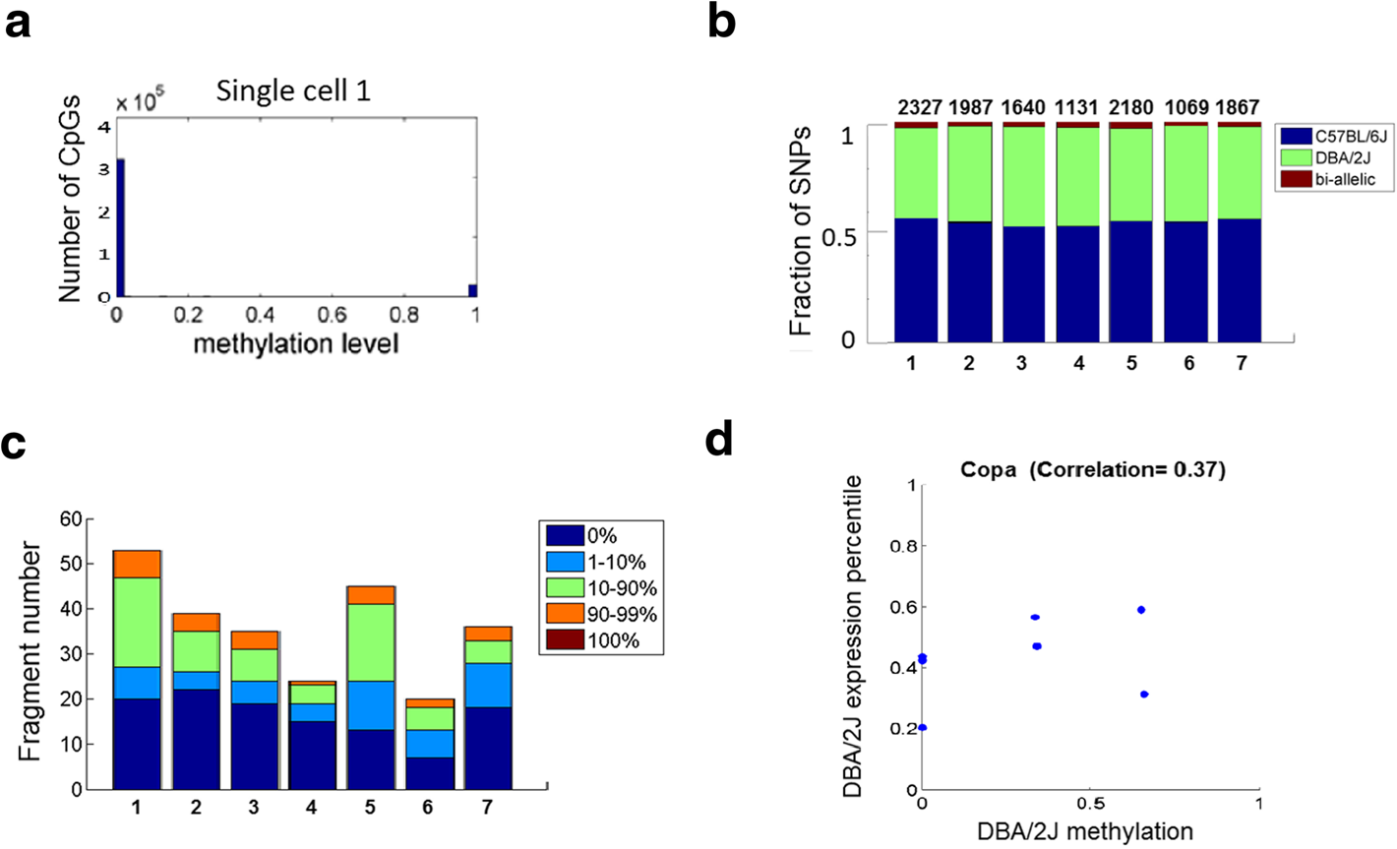
Profile of allelic-specific transcription and methylation. **a** *Histogram* of methylation levels for all CpG sites within a representative single cell. **b** *Bar graph* showing the proportion of mono-allellic or bi-allelic SNPs as measured by scRRBS. Each site with known strain-specific SNPs that overlapped with RRBS fragments were interrogated for their presence of C57BL/6 J and DBA/2 J SNPs. Sites that covered both SNPs were considered bi-allelic otherwise are considered mono-allelic. Each *bar* represents the distribution for a single cell. **c** *Bar graph* showing the distribution of methylation level within bi-allelic fragments. Each *bar* represents the distribution for a single cell. **d** *Scatter plot* of DBA/2 J-strain-specific *Copa* methylation and expression across single cells. Each *point* represents a single cell and the position on the graph shows the DBA/2 J specific methylation and expression levels for *Copa*

Theoretically, there are only three possible levels for the methylation of a CpG site in a diploid single cell, which are 1 (both alleles methylated), 0 (both alleles unmethylated), and 0.5 (only one of the two alleles methylated). Our sequencing results showed that 95–98 % of the CpG sites detected are within these three possibilities (Fig. 5a), indicating the vast majority of assayed CG sites are accurately digitized. This distribution is similar to previous single-cell methylation analysis results [12, 13]. However, it is unclear whether the bimodal CpG methylation distribution accurately represents one or two alleles. In a subset of data presented in this paper, we have used a hybrid F2 generation mouse for a number of experiments (F2 generation by backcrossing a F1 female C57BL/6 J × DBA/2 J with male C57BL/6 J). Although the DBA/2 J SNP number in F2 is underrepresented compared to the F1, we still were able to leverage SNP information for downstream study. Leveraging our single-base resolution of bisulfite sequencing, we detected differential SNPs between the two strains to estimate the level of allelic representation. In total, we found approximately 2000 RRBS fragments contained informative SNPs (fragments that are expected to contain SNPs from both C57BL/6 J and DBA/2 J strains). However, our analysis indicated that only a small fraction exhibited representation from both mouse strains. Thus the vast majority of assayed CpGs represent only one of two possible alleles (Fig. 5b). Interestingly, bi-allelic RRBS fragments tend to have greater non-bimodal states, indicating true differences in allelic methylation (Fig. 5c). Nonetheless, bi-allelic fragments still showed a majority in a hypomethylated state, consistent with the overall target regions captured by RRBS.

We next examined the correlation of allelic methylation with allelic expression patterns. Because our F2 hybrid mouse contains regions where C57BL/6 J can be bi-allelic, we only examined the influence of maternally derived DBA/2 J SNPs on DBA/2 J transcripts. Only highly expressed genes were considered for analysis to ensure sufficient SNP coverage. In this proof-of-principle analysis, we detected between 5 and 30 genes per cell that are covered by DBA/2 J SNPs in both scRRBS and scRNA-seq fractions, depending on coverage of the respective libraries. Nonetheless, using this method we were able to find correlations between DBA/2 J specific gene body methylation and its effect on gene expression (Fig. 5d). This method provides a potential way to discover the correlation of allelic-specific methylation and gene transcription by using the SNP information at single-cell level.

## Discussion

In this study, we established a method to simultaneously profile both the transcriptome and DNA methylome from the same DRG neuron. We investigated the correlation of mRNA transcription with DNA methylation in either promoter or gene body at a single-allele level within single cells. We conclude that gene activity can be more reliably predicted using either gene promoter or gene body methylation based on the CpG content of the promoter. Specifically, promoter methylation is inversely correlated with non-CGI promoter genes and gene body methylation is positively correlated with genes containing CGI promoters.

Previously, based on transcriptome and methylome analysis of bulk rat DRG cells, Hartung et al. [20] found that high CpG density promoter are consistently hypomethylated while the corresponding gene body are differentially methylated between high and low expressed genes. In a separate study using the DRG injury model in rats, thousands of CpG sites were reported to be differentially methylated, but a minimal number were associated with changes in gene expression [21]. The discrepancy between these two published studies may be due to the differences in sample preparation of bulk tissues. The DRG represents heterogeneous population of neuronal and glial cells, in which sensory neurons are further divided in many subtypes based on marker gene expression (e.g. TrkA, TrkB, and TrkC expression). More recently, the DRG neuronal cells are further subdivided into 11 subtypes based on single-cell RNA transcriptome analysis [11]. Thus, the previous bulk studies are susceptible to variance due to differences in subtype representation during sample collection. In addition, any meaningful differences between subtypes may be masked in bulk preparations.

We observed positive correlation of gene body methylation with gene expression for those genes with CGI promoters but not non-CGI promoter at the single-cell resolution. Recently, based on meta-analysis of genome-wide methylation, mRNA expression, and chromatin modifications, Jjingo et al. suggest that gene-body methylation levels are predominantly shaped via the accessibility of the DNA to methylating enzyme complexes [22]. Our current study shows that gene expression level of CGI promoter genes is higher than non-CGI promoter genes in single DRG neurons (Additional file 1: Figure S11, t test, *p* <10^−4^), consistent with this hypothesis. In addition, Karlic reported that different histone modifications can be used to predict the gene expression driven by high CpG content promoters (HCP) or low CpG promoters (LCP). They found that H4K20me1 are enriched in HCP gene body but not in LCP gene body [23]. These findings implicate that histone modification may influence the accessibility of the DNA to DNA methyltransferase complexes, leading to different correlation of gene body methylation with gene expression for CGI versus non-CGI promoter genes.

The number of genes detected by scMT-seq (around 10,000) is comparable with the coverage achieved by traditional Smart2-seq using single-cell soma. Although we found that a small set of genes that are more enriched in soma, these genes only account for 0.03 % of all the genes detected. These results are consistent with another study comparing cytosolic, nuclear, and soma RNA fractions [24]. Although they identified 192 genes that are unique to the neuronal nucleus, none of these genes overlapped with genes specifically expressed in soma compared to cytosol in our dataset. This could be explained by nuclear RNA representing only a tiny fraction of entire cell body RNA [25].

While this study is in revision, Angermueller published a method named “scM&T” to analyze transcriptome DNA methylome for single cells [26]. Compared to their method of methylome analysis via whole genome bisulfite sequencing [26], our method via scRRBS is a well-established protocol for being cost-effective and reliable in covering CGIs and other CpG regions blanked by the MspI (CCGG) restriction site. Indeed, even with low sequencing depth, our results showed similar level of overlap of CGI with scM&T [12, 26]. More recently, Hou et al. reported a similar method named scTrio-seq based on scRRBS to detect transcriptome and methylome for single cells through physical separation of RNA and nucleus [27]. While the conclusions between their study and ours are largely similar, we find that scTrio-seq has a much lower transcriptome coverage (6200 vs. 9900), likely due to major different experimental approaches to isolate cytosol RNA. Nonetheless, both methods provide a simple and cost-effective way to isolate DNA and RNA for integrated methylome and transcription analysis.

Our current scMT-seq method has several limitations that should be overcome with future technology. For example, scRRBS only covers approximately 1 % of CpG sites across the whole genome, while single-cell whole genome bisulfite sequencing could cover up to 48.4 % of CpG sites of the whole genome [12], enabling more comprehensive analysis of DNA methylation and RNA transcription. Another limitation of our method is a high rate of allele drop-out, making it less suitable for analysis of those genes that are differentially expressed between alleles due to differential methylation. Improvements in the following aspects could improve the coverage of methylation detect of both alleles: optimize the bisulfite treatment condition to reduce the degradation of DNA as well as the purification methods to reduce the stochastic loss of DNA, and improve the adapter ligation efficiency to capture more DNA fragments.

## Conclusion

Integrating DNA methylome and transcriptome analysis would provide a direct correlation between DNA methylation and gene transcription. By developing the current scMT-seq method, we achieved simultaneous profiling of transcriptome and DNA methylome from a single neuron. Our integrated analysis shows that methylation of non-CGI promoters is better anti-correlated with gene transcription while gene body methylation of CGI promoter genes is better correlated with gene transcription. Our results lay a solid foundation to study epigenetic mechanism underlying neuronal gene expression at a single-cell level.

## Methods

### Animals and isolation of DRG neurons

Animals were kept in cages under 12-h light–dark conditions. In this study, we used several strains of adult mice for technology development including 129/B6 outbreed or F1 hybrid (C57BL/6 J × DBA/2 J [B6/D2]) or F2 hybrid mice (F1 female B6/D2 mice backcrossed with C57BL/6 J [B6] males). Adult lumbar DRGs (L4, L5) were dissected and dissociated with trypsin according to a published protocol [28]. After being dissociated into single cells, samples were incubated in DMEM medium containing 10 % FBS.

### Isolation of nucleus and cytoplasma from a single DRG neuron

Single cells were picked by using micro-capillary pipette under microscope. Single cells were incubated in a drop of cell membrane-selective lysis buffer (2 % Triton, 20 mM NaCl, and 20 mM Tris, 2 U/uL RNase inhibitor, 1:40,000 ERCC) [29], which was on the wall of a PCR tube. After incubation for 5 min, the cell membrane was lysed thoroughly and the cell nucleus was exposed. The nucleus was picked by a micro capillary pipette in 0.2 μL buffer and transferred into another PCR tube containing 4 uL RRBS lysis buffer. A total of 1 μl oligo-dT primer (10 μM) and 1 μ dNTP (10 mM) were added into the tube including cytosol RNA. After briefly centrifuging, the tubes containing nucleus and cytosol, respectively, were put on dry ice immediately, and transferred to −80 °C until the next step.

We used 50 DRG single cells to isolate DNA and RNA. Forty-four of the 50 (88 %) RNA-seq libraries passed quality check after sequencing. However, for the DNA fraction, only 15 of 22 (or 68 %) libraries constructed passed quality filter after sequencing. Major sources of failure among scRRBS appear to be no amplification (did not show bands after PCR) or lower library complexity.

### Merge-and-split experiments

To test the technical variance of micropipette, cytosols of two individual cells were merged together and split into two equal parts by micropippette. Briefly, two single cells were transferred to 4 μL lysis buffer and incubated for 5 min. After picking out the two nuclei, the rest of the solution was mixed and split into two tubes by micropipette. Libraries were made by the following protocol and sequenced on the Illumina Mi-seq machine following manufacturer’s specifications.

### Single-cell RNA-seq library construction

Single-cell complementary DNA was amplified from the tubes containing cytosol according to the Smart2-seq protocol. Instead of using Superscript II, we used Superscript III for reverse transcription. After amplification and purification, 0.1 ng cDNA was used for Nextera Tagmentation and library construction. Library quality was assessed using Agilent Bioanalyzer 2100.

### Single-nucleus (cell) RRBS library construction

Single-nucleus (cell) RRBS libraries were constructed according to a previously published method with some modification [19]. Briefly, a single nucleus isolated from a single DRG cell was put into lysis buffer, and double-strand DNA was released and digested by MspI along with spike-in lambda DNA. After end-repairing and dA tailing, DNA fragments were ligated with adaptors, then subjected to bisulfite conversion. Following that, converted DNA was purified and enriched by two rounds of PCR amplification. To reduce the PCR products from adapters, we optimized the PCR cycle number to 20 cycles and 12 cycles for the first and second rounds, respectively. Libraries between 180 bp and 500 bp were selected by page gel and purified for deep sequencing in Illumina Hiseq 2500 machines.

### RNA-seq analysis

Raw reads from library sequencing were mapped to the mouse (mm10) genome using default parameters in STAR aligner [30]. Reads that failed to map to the genome were re-mapped to their respective mRNA sequences to capture reads that span exons. Only reads that were uniquely aligned were retained. Data normalization was performed by transforming uniquely mapped transcript reads to RPKM using a previous established pipeline [15]. Genes with low expression (average RPKM <0.1) were filtered out, followed by quantile normalization. Samples were excluded based on a variety of quality assessments. Libraries with poor alignment (<20 %) and poor gene coverage (<3000 genes with RPKM >1) were excluded. Clustering analysis and PCA analysis were performed by using built-in functions in Matlab.

Differential expressed analysis between cytosol and soma was implemented in DESeq [31]. Genes that are not expressed in any samples were not taken into consideration. For each gene, DESeq reports its mean read count in cytosol, soma, and the adjusted *p* value testing for differential expression. These mean counts were plotted and those genes under threshold of *p* <0.01 were significantly differentially expressed between cytosol and soma and marked as three red dots in Fig. 1c.

### Methylation analysis

Raw reads for the scRRBS libraries were mapped to the mouse (mm10) genome using the default parameters in BS-seeker2 for RRBS mapping. Methylation calling was performed as previously described [32]. CpG sites that were covered by more than 1 or 5 reads were counted, respectively. To evaluate whether the variance we observed at individual CpG sites is greater than what would be expected from the entire population of CpGs across all samples (null distribution), we used a test of variance, also known as the F-test, and performed multiple-testing using the Benjamini–Hochberg method. For promoter methylation calculation, CpG sites that are located 500 bp upstream of the transcription start site (TSS) were counted; methylation level for promoter (with more than 5 CpG sites detected) and gene body region (with CpG sites more than 0.5 kb detected) were calculated by using bedtools package.

### Gene transcription and methylation level correlation analysis

Transcription level (RPKM) was transformed into percentile rank. Correlation of transcription and methylation was calculated by Pearson correlation in R. The average methylation level for promoter and gene body detected was calculated and its correlation with corresponding transcription was examined for those genes detected in more than five cells.

### SNP analysis

RNA-seq data of single cells derived from F2 hybrid mice (offspring of F1 female [DBA/2 J × C57BL/6 J] backcrossed with male B6) was subjected to SNP analysis. DBA/2 J annotated SNPs were downloaded from the Wellcome Trust Sanger Institute (dbSNP142). SNP calling followed the GATK Best Practices guideline (version 3.5). Briefly, raw reads were mapped to the mm10 genome using STAR aligner using default parameters followed by base quality scores recalibration. SNP calling was performed jointly for all 15 cytosol samples using the HaplotypeCaller function with default parameters. Only annotated SNP hits with QD score greater than 20 and FS score less than 60 were accepted for downstream analysis. DBA/2 J allelic expression was estimated by taking the average allelic balance across all SNPs within a gene then multiplied by the genes overall RPKM.

SNP calling in RRBS libraries were done by traversing pileups of RRBS fragments with the DBA/2 J SNP reference. C-T SNPs were ignored and only SNPs with coverage of 5 reads were accepted.

### Availability of data and material

All the related data can be downloaded from GEO with the accession number GSE76483.

### Ethics

All the procedures are performed according to institutional guidelines and approved by animal research committee of UCLA (protocol 2001-045-41).

## Additional file

Additional file 1: Contains Supplementary Figures 1–11 and Supplementary Table 1. (PDF 1536 kb)

## Abbreviations

CGI: CpG island
DRG: Dorsal root ganglion
scMT-seq: single cell methylome and transcriptome sequencing
