# Supplementary material for: Simultaneous profiling of transcriptome and DNA methylome from a single cell
Source: Genome Biol. 2016 May 5;17:88. doi: 10.1186/s13059-016-0950-z (PMC4858893; doi:10.1186/s13059-016-0950-z)
Supplement: Additional file 1: — Contains Supplementary Figures 1–11 and Supplementary Table 1. (PDF 1536 kb) [file 13059_2016_950_MOESM1_ESM.pdf]

Additional file 1:

Supplementary materials

Simultaneous profiling of transcriptome and DNA methylome from a single cell

Youjin Hu[1, 2†], Kevin Huang[1, 3†], Qin An[1], Guizhen Du[1], Ganlu Hu[2], Jinfeng Xue[2], Xianmin Zhu[2], Cun-Yu Wang[3], Zhigang Xue[2, 4*], and Guoping Fan[1*]

1. Department of Human Genetics, UCLA, USA
2. Tongji Hospital, School of Medicine, Tongji University, China
3. Department of Oral Biology, School of Dentistry, UCLA, USA
4. Suzhou Institute of Tongji University, Jiangsu Province, China

<u>**Contents**</u>

Figure S1. Single cell cytosol RNA sequencing has highly technical reproducibility.

Figure S2. Analysis of technical variations between single cytosol- and soma- RNA sequencing.

Figure S3. Saturation analysis of scRRBS libraries.

Figure S4. Relationship between CpG methylation variance and FDR.

Figure S5. Distribution of variably methylated CpGs across the genome.

Figure S6. Correlation of DNA methylation with gene transcription in a single cell.

Figure S7. Simultaneous detection of gene transcription and CGI promoter methylation in a single cell.

Figure S8. Simultaneous detection of gene transcription and non-CGI promoter methylation in a single cell.

Figure S9. Consistent levels of promoter hypomethylation and gene expression across multiple samples.

Figure S10. Simultaneous detection of gene transcription and gene body methylation in a single cell.

Figure S11. Comparison of gene expression levels in those genes carrying either CGI or non-CGI promoters.

Table S1. Methylome analysis for single soma across datasets

Figure S1. Single cell cytosol RNA sequencing has highly technical reproducibility.
A) PCA analysis for the “merge-and-split” experiment on 9 pairs of cytosols from single-cells. For each pair of cytosol, cytosolic RNA were merged then split by into two fractions using micropipette. Each pair is assigned a unique letter and color as indicated by the legend. For example, A1 and A2 are the two replicates for pair A; B1 and B2 are the two replicates for pair B, etc. **B**) Heatmap showing correlation of ERCC spiked-in RNA between single cells. Pearson correlation was calculated based on the RPKM of the spiked-in ERCC content. In the top color bar, blue indicates cytosol while red indicates soma.

A

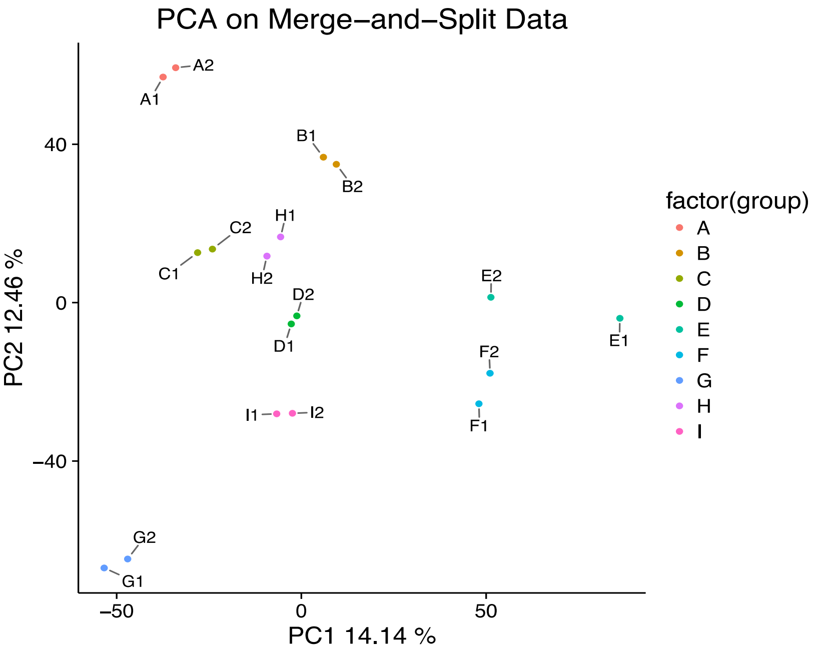

B

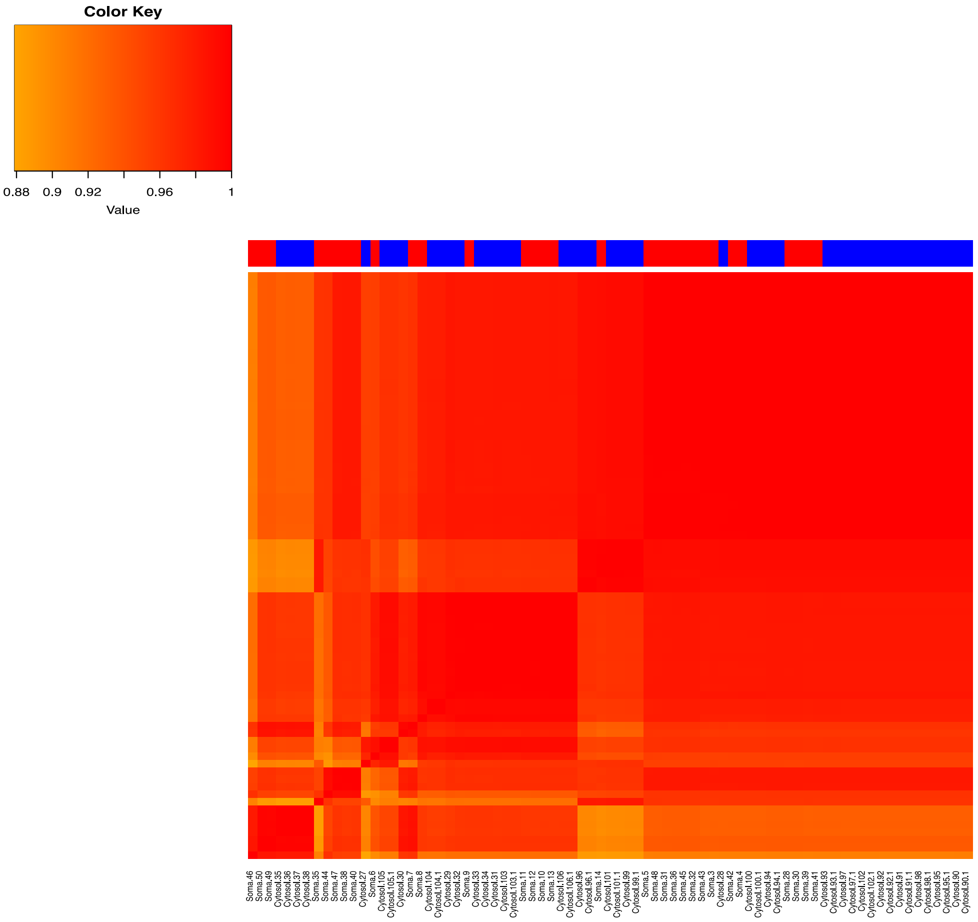

Figure S2. Analysis of technical variations between single cytosol- and soma- RNA sequencing.

Scatter plots showing the relationship between read depth and coefficient of variance (CV). Each dot represents a single gene and its position on the plot represents the genes' average depth across samples (y-axis) and its coefficient of variance (x-axis). Solid line represents the best fit line of the data and dotted lines represent the upper and lower bound for 95% confidence intervals. Red lines represent cytosol-cytosol samples only and blue lines represent soma-soma samples only. The data indicate the cytosol RNA-seq technical variation is similar to soma RNA-seq.

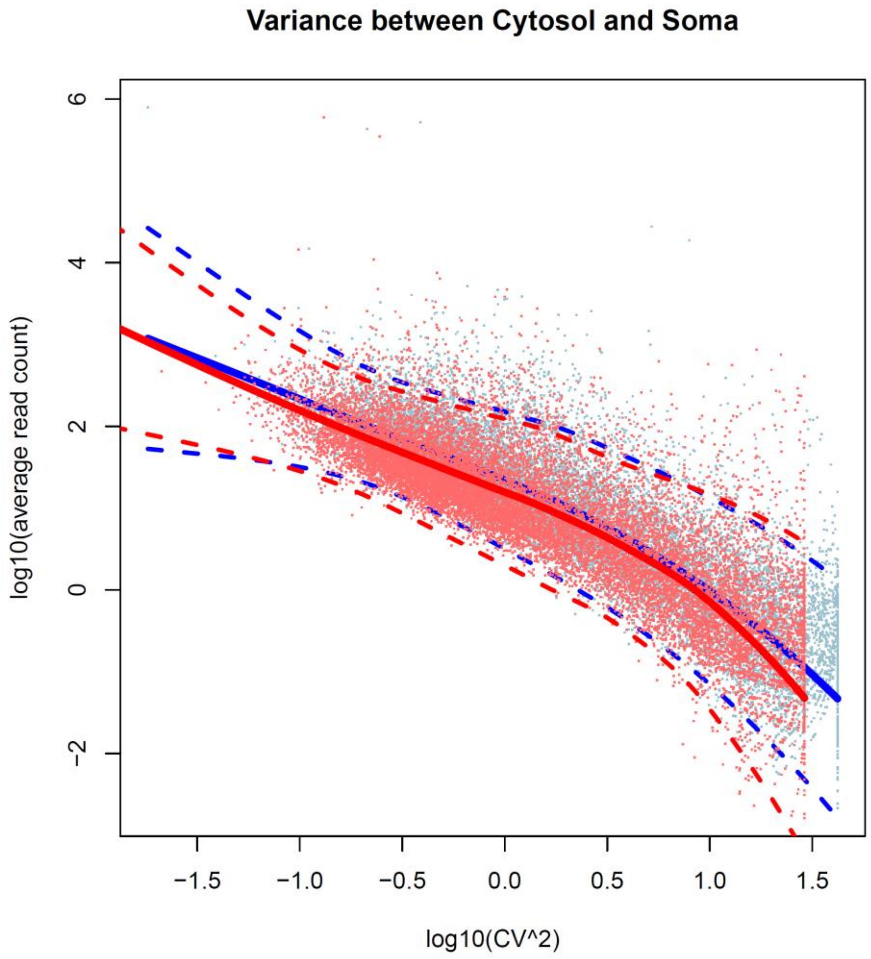

Figure S3. Saturation analysis of scRRBS libraries.
Line plots of sequencing depth and CG coverage (>=5 reads) for scRRBS libraries. With the increase of the sequencing depth, more CpG sites were captured but saturation was reached around 6 million reads for majority of samples.

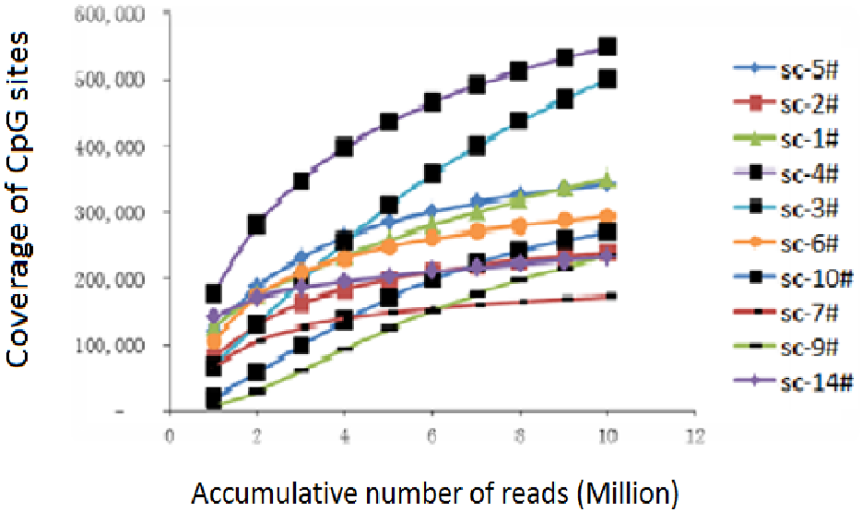

Figure S4. Relationship between CpG methylation variance and FDR.
We plot the *FDR* (estimated by Benjamani-Hochberg correction of the F-statistic) against the variance for each CpG methylation that is shared across at least 8 samples. Red dotted line shows the cutoff of FDR = 0.05. By random chance, the expected variance between shared CpGs would be within the range of 0.02 to 0.14 (FDR>0.05). We identified 6,800 CpGs that have significantly greater variance than 0.14 and *FDR* <0.05.

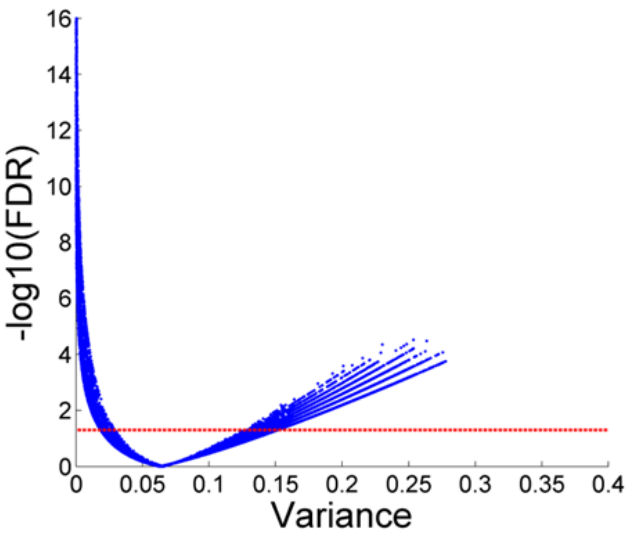

Figure S5. Distribution of variably methylated CpGs across the genome.
Pie chart showing the distribution of 6,800 variably methylated CpG sites for different genomic features as indicated. Enrichment of genomic features compared to background is shown in Figure 2D.

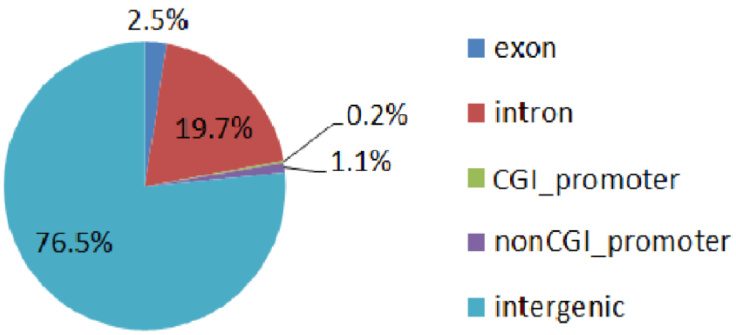

Figure S6. Correlation of DNA methylation with gene transcription in a single cell. We bin the genes from 5' to 3' into deciles (x-axis) and calculate their individual contributions to Pearson correlation with gene expression. These data show that promoter methylation is inversely correlated with gene expression, whereas gene body methylation tends to have weak correlation with expression. The negative correlation at promoters may be due to different definitions of the promoter. In our promoter analysis, we strictly used -500bp from the TSS. However, this figure shows the negative correlation is most distinct at the first 5' decile within the gene body.

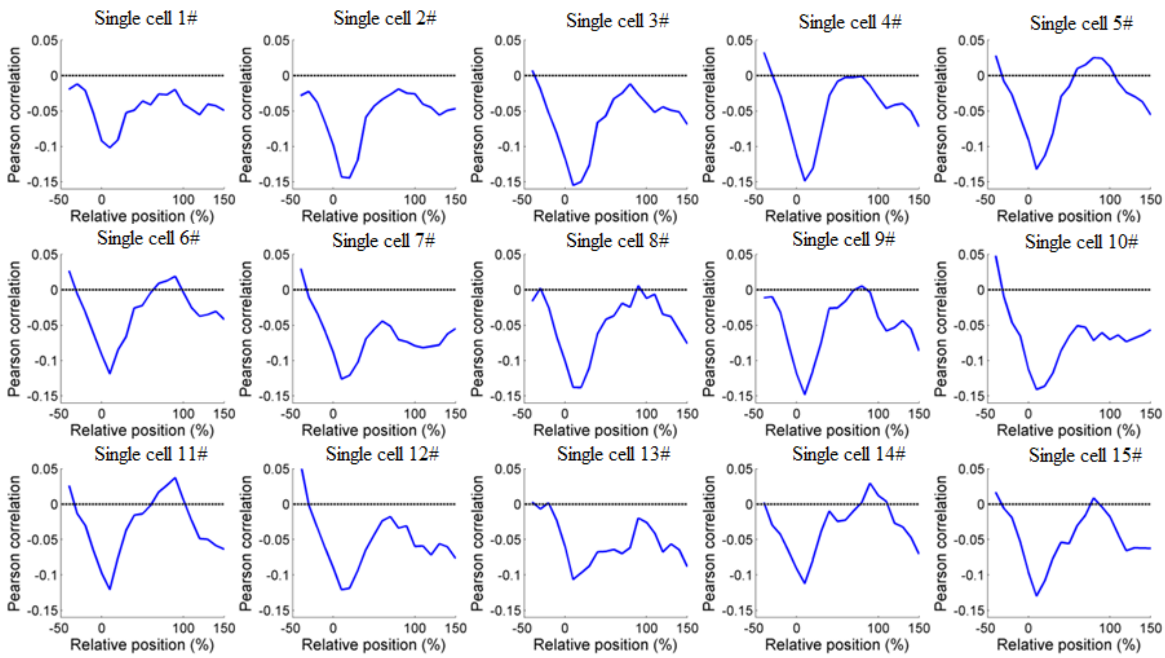

Figure S7. Simultaneous detection of gene transcription and CGI promoter methylation in a single cell.

Each panel shows a scatterplot where each point represents a gene, and the position on the plot represents the gene's methylation at the proximal promoter (-500bp to TSS) and expression rank (y-axis). Pearson correlation of gene transcription and promoter methylation level is indicated on the title of each panel.

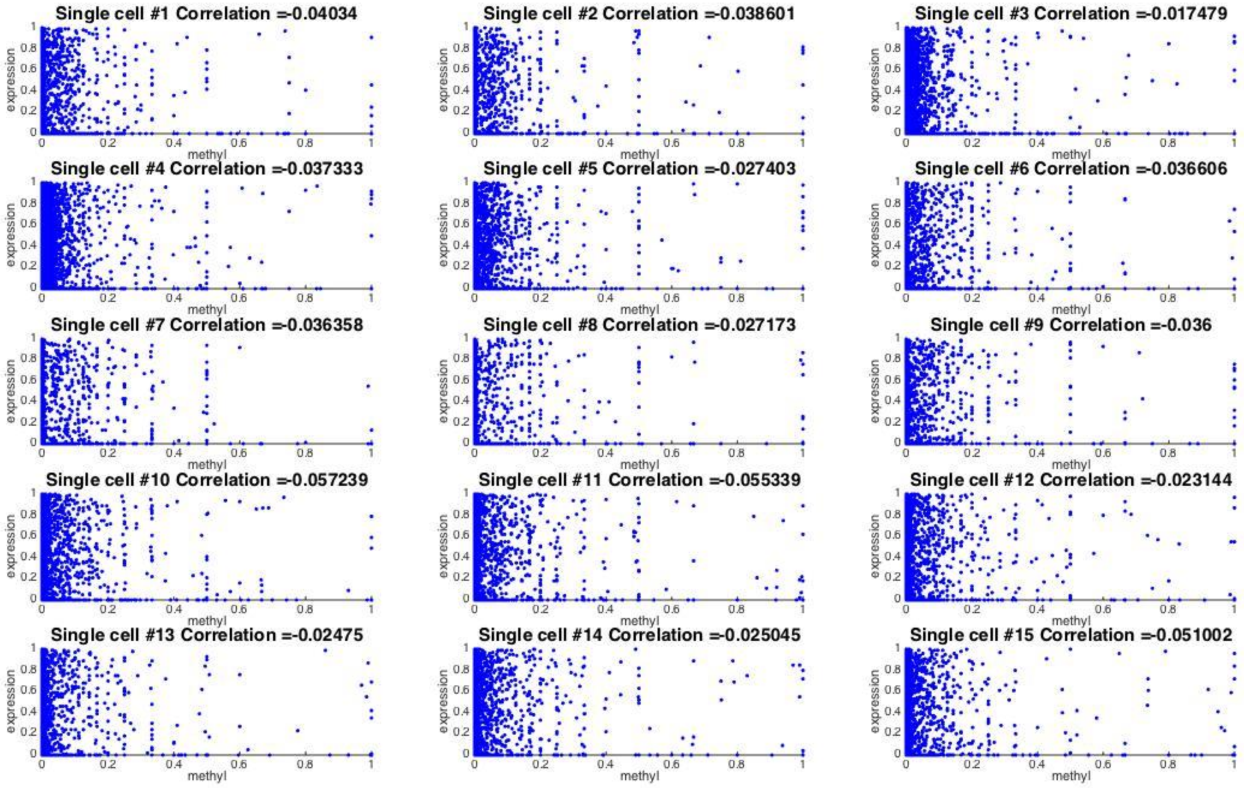

Figure S8. Simultaneous detection of gene transcription and non-CGI promoter methylation in a single cell.

Each panel shows a scatterplot where each point represents a gene, and the position on the plot represents the gene's methylation at the proximal promoter (-500bp to TSS) and expression rank (y-axis). Pearson correlation of gene transcription and promoter methylation level is indicated on the title of each panel.

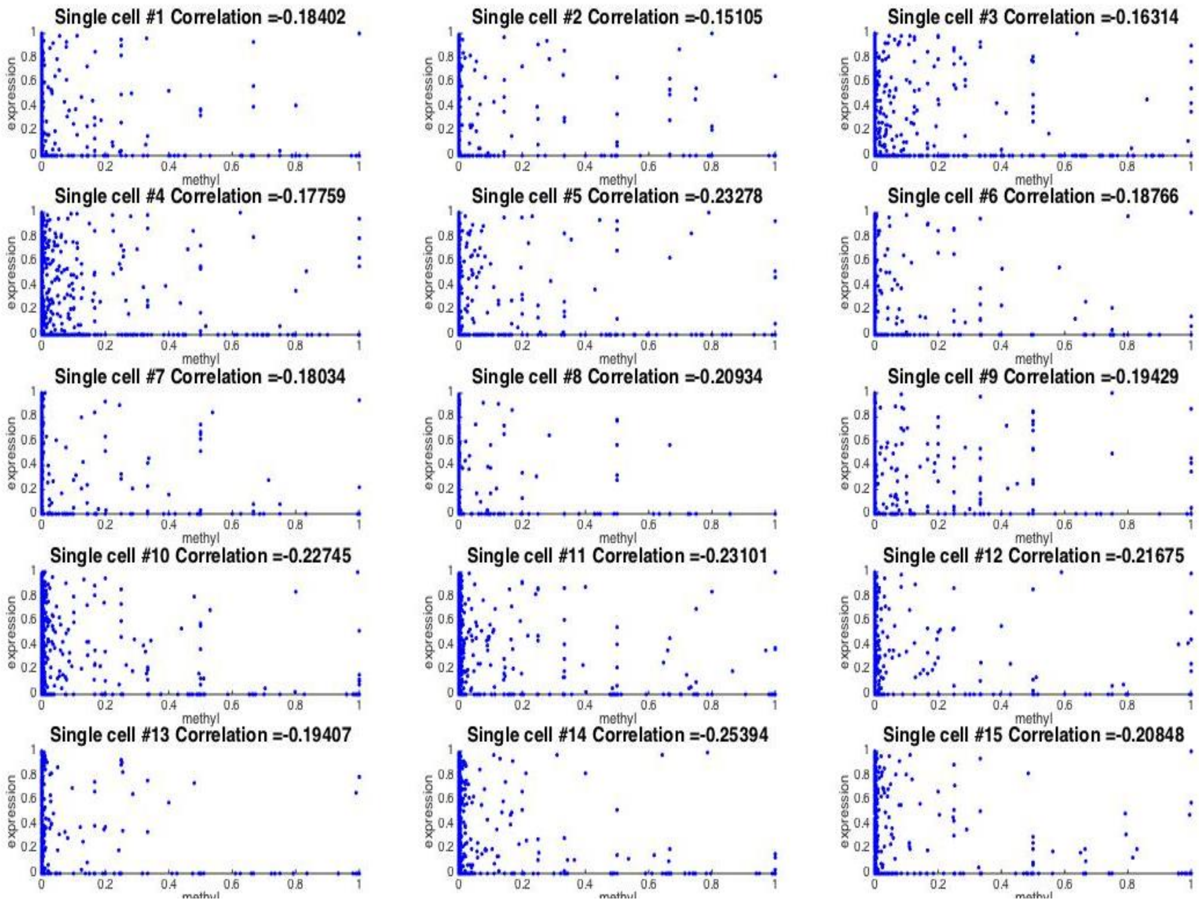

Figure S9. Consistent levels of promoter hypomethylation and gene expression across multiple samples.

Scatter plots showing representative genes with stable promoter hypomethylation with concurrent high expression (**A**) or low expression (**B**). Each point represents a single-cell, and the position on the graph indicates the average promoter methylation level (x-axis) and expression rank (y-axis).

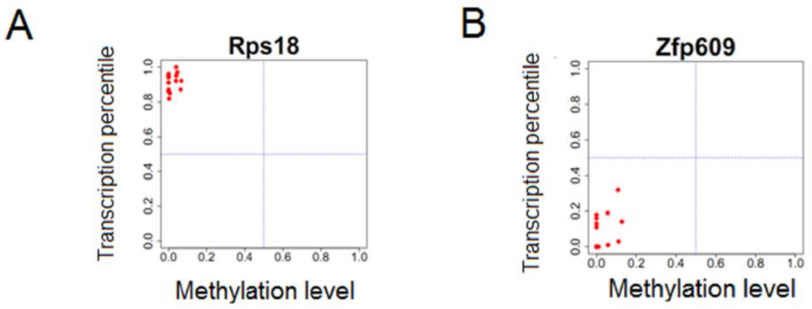

Figure S10. Simultaneous detection of gene transcription and gene body methylation in a single cell.

Each panel shows a scatterplot where each point represents a gene, and the position on the plot represents the gene's methylation across the gene body (with a minimal coverage of 1 CpG per 2kb) and expression rank (y-axis). Pearson correlation of gene transcription and gene body methylation level is indicated on the title of each panel.

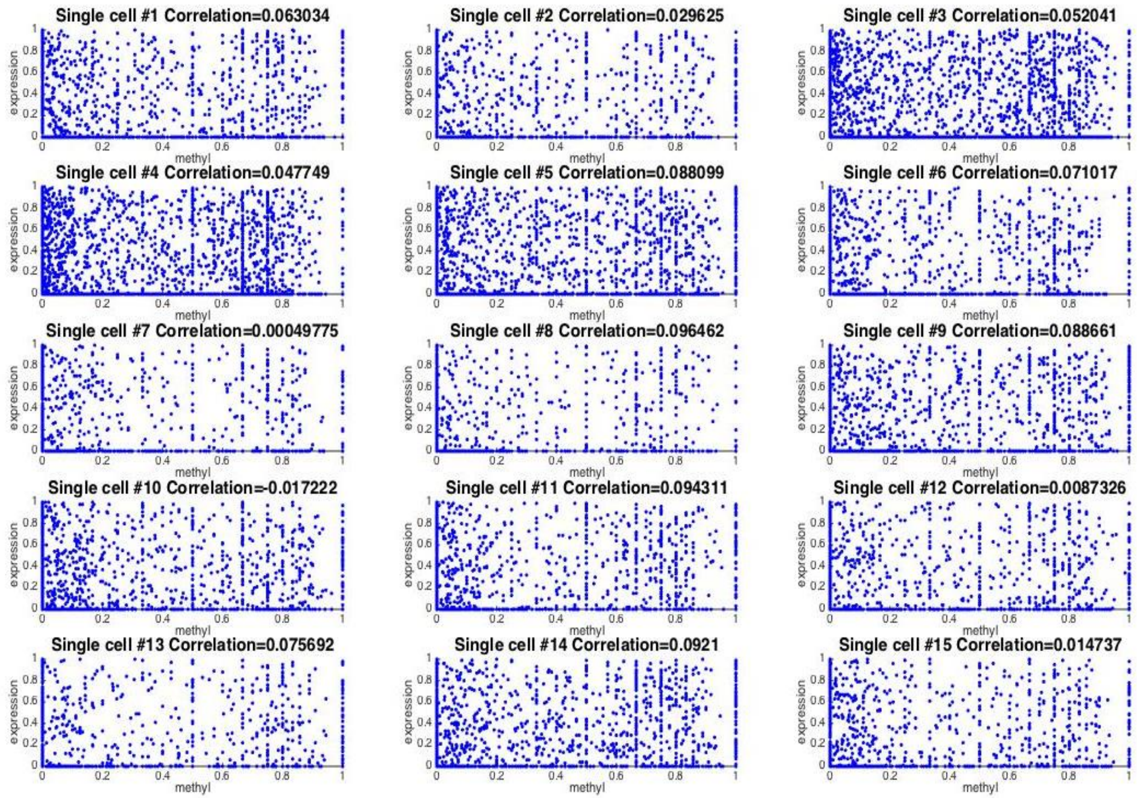

Figure S11. Comparison of gene expression levels in those genes carrying either CGI or non-CGI promoters.

Red boxplot shows the distribution of gene expression for the CGI promoter genes and blue boxplot shows the distribution of gene expression of non-CGI promoter genes.

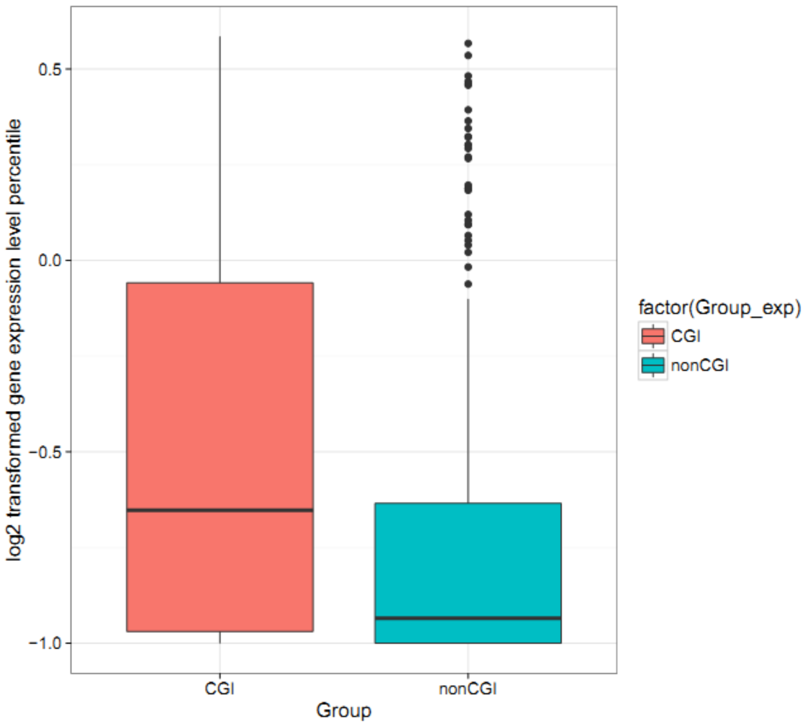

Table S1. Methylome analysis for single soma across datasets

Samples	Data set	CpG(5x)	Conversion rate
scRRBS_mESC_Single_Cell1	Guo et al[1]	342,121	99.59%
scRRBS_mESC_Single_Cell2	Guo et al[1]	238,120	99.57%
scRRBS_mESC_Single_Cell3	Guo et al[1]	498,910	99.64%
scRRBS_mESC_Single_Cell4	Guo et al[1]	547,785	99.48%
scRRBS_mESC_Single_Cell5	Guo et al[1]	350,744	99.77%
scRRBS_mESC_Single_Cell6	Guo et al[1]	293,706	99.63%
scRRBS_mESC_Single_Cell7	Guo et al[1]	231,743	99.94%
Single DRG cell_soma	Hu et al (this study)	270,278	99.30%

References:

1. Guo H, Zhu P, Guo F, Li X, Wu X, Fan X, *et al*. Profiling DNA methylome landscapes of mammalian cells with single-cell reduced-representation bisulfite sequencing. Nat Protoc. 2015; 10**:**645-659.
